# Safe Sexual Behavior Intentions among College Students: The Construction of an Extended Theory of Planned Behavior

**DOI:** 10.3390/ijerph18126349

**Published:** 2021-06-11

**Authors:** Chien-Liang Lin, Yuan Ye, Peng Lin, Xiao-Ling Lai, Yuan-Qing Jin, Xin Wang, Yu-Sheng Su

**Affiliations:** 1 College of Science and Technology, Ningbo University, Cixi 315211, China; linjianliang@nbu.edu.cn (C.-L.L.); yeyuan@kmvs.khc.edu.tw (Y.Y.); linpeng@kmvs.khc.edu.tw (P.L.); laixiaoling@kmvs.khc.edu.tw (X.-L.L.); jinyuanqing@nbu.edu.cn (Y.-Q.J.); wxin@nbu.edu.cn (X.W.); 2 Department of Computer Science and Engineering, National Taiwan Ocean University, Keelung 20224, Taiwan

**Keywords:** sexual health education, college students, safe sexual behaviors intention, Theory of Planned Behavior

## Abstract

Sexual health education is an essential part of quality-oriented education for college students. It aims to help these students to acquire knowledge of sexual physiology, sexual psychology, and sexual social norms that is consistent with the maturity of the students. Along with college students’attitudes toward sex, their perceptions regarding sexual behavior have also undergone profound changes. The importance of safe sexual behavior, sexual taboos, and sexual autonomy are gaining increasing attention as Chinese society is becoming more open. For college students who have just reached adulthood and have full autonomy of themselves, however, are they really going to have sexual behavior without careful consideration? Or is it something they have planned to do in the first place? To answer the above questions, this study was conducted to understand the relationship between college students’ attitudes toward sex, subjective norms, and behavioral control of their sexual behavior intentions by applying the Theory of Planned Behavior. In this study, 460 valid questionnaires were collected from Chinese college students and analyzed with partial least squares structural equation modeling (PLS-SEM). This study analyzes the relationship of multiple factors, including those influencing college students’ sexual behavior intentions. Meanwhile, it also compares the differences in factors affecting sexual behavior intentions between college students with or without sexual experience and those of different genders. Based on the results of the study, it was found that, first, subjective norms and perceived behavioral control of college students had a significant effect on safe sexual behavior intentions, while attitudes did not have a significant effect on safe sexual behavior intentions. Second, the gender and sexual experience of college students had a significant effect on safe sexual behavior intentions. Third, non-sexually experienced college students were more likely to be influenced by external factors. Relevant future research suggestions will be proposed based on the results of this study. Finally, this study helps to provide substantive suggestions for enhancing safe sexual behavior among college students in the context of universal higher education, as well as strengthening the self-protection of college students and providing practical advice for the development of sex education in China.

## 1. Introduction

Sexual health is a topic that attracts worldwide attention. According to the World Health Organization, sexual health involves the physical, mental, spiritual, and social levels of comfort associated with sex. Everyone should hold a respectful and positive attitude toward sexual relations. Moreover, sexual experiences should be safe and enjoyable as well as free from pressure, discrimination, and violence; in addition, the right to sexual health should be actively protected [1]. According to the *China Statistical Yearbook*, the population aged 19–24 years in China has reached 80 million [2]. China’s economic transformation and opening to the outside world have had a tremendous impact on the life of college students in the country. People’s access to education has continually improved, with the number of college students increasing from 5.6 million in 2000 to 30.3 million in 2019 [3]. The popularity of the Internet and social media exposes young people to diverse cultural and social norms and values related to sex and marriage [4]. Such values can sharply contrast traditional Chinese values, especially those regarding women [5,6]. Along with contemporary societal changes, the problems caused by premarital sex among college students are also increasing. According to an anonymous survey conducted by Sung et al. [7] among college students in 18 Chinese provinces, a greater proportion of men than of women had engaged in sexual behavior, and more than half of college students who had engaged in sexual activity had not employed appropriate protective measures. Lyu et al. [8] and Chang et al. [9] reported, respectively, that 18.7% and 16% of Chinese college students had previous sex experience, indicating an annually increasing trend.

In China, sexual health education for contemporary college students has been extremely conservative, and many college students avoid talking about sex-related topics. Most parents also do not provide positive sexual health education to their children [10]. With the rapid proliferation of the Internet, the sexual cultures of countries worldwide have also evolved. As the sources of sexual knowledge have diversified, social health problems have also emerged, such as human immunodeficiency virus (HIV) infection, abortion, and pregnancy out of wedlock. [11]. According to statistics from the Chinese Center for Disease Control and Prevention [12], from January to August 2020, the number of acquired immunodeficiency syndrome (AIDS) cases in China was 39,349, and the number of related deaths was 11,595. These numbers have tended to increase annually; moreover, people infected with AIDS are becoming younger, and the incidence of AIDS among college students is constantly increasing. Liu [13] revealed that in 2003, 7.215 million legal abortions were performed in China, and by 2015, this number had increased to more than 13 million. According to *China Youth Daily* [14], more than half of the women undergoing abortions in China are under 25 years old, and college students have become the driving force behind this rise in numbers. Therefore, to reduce the social problems caused by unsafe sexual behaviors among college students, first examining the understanding of Chinese college students regarding safe sexual behaviors is essential; relevant data may help to promote the popularization of sex education in China’s universities in the future.

Previous studies on people’s psychological and social behavior, especially studies concerning health issues, have indicated that people’s engagement in any activities is planned behavior [15]. According to previous studies, people’s attitudes, subjective norms, and perceived behavioral control affect their behavioral intentions and thus change their behaviors. College students may acquire incorrect knowledge about sex, which may affect their beliefs, attitudes, subjective norms, and behavioral intentions regarding sexual behavior, and subsequently affect their social development and behavior [16]. Research by Armitage and Tailbudeen [17] revealed that 46% of the variance in the intention to have safe sex could be explained by subjective norms, attitudes, and perceived behavioral control. Fishbein and Ajzen [18] argued that human behavior is affected by diverse multidimensional background factors. Personal (e.g., personality traits, values, stereotypes, perception of risk, and experience), social (e.g., educational background, age, sex, religion, ethnicity, and culture), and informational (e.g., knowledge, media, and interventions) factors are the main background factors. Therefore, discussions of safe sexual behavior require considering more than just individual attitudes toward safe sexual behavior, subjective norms, and the ability to control behavior. It is necessary to include variables such as gender or sexual experience between individuals to explain the effects of safe sex on college students. Throughout the above arguments, the research question posed in this study is: Do gender- and experience-specific college students’ attitudes, subjective norms, and behavioral control about safe sex behavior affect their sexual intentions?

Based on these questions, this study collected relevant data from Chinese college students through a questionnaire survey. We explored factors influencing Chinese college students’ sexual behavior intentions through a structural equation model to provide a theoretical basis for future research on Chinese sexual behavior. This study analyzed Chinese college students’ perceptions of safe sexual behavior and identified the factors that influence college students’ perceptions of safe sexual behavior. The findings can provide substantive suggestions for future and practical advice to promote the popularization of sex education in Chinese colleges and universities.

## 2. Literature Review

The Theory Planned Behavior (TPB), proposed by Ajzen [19], is used to analyze the factors affecting intention and to predict behavioral intentions, both of which are valuable to research. The TPB includes three variable factors that affect sexual behavior intentions: perceived behavioral control (subjective assessment of the degree of promotion of or obstruction to performing safe sexual behaviors), attitude (positive or negative evaluations of safe sexual behaviors), and subjective norms (the degree to which individuals perceive the positive or negative support of significant others or reference groups regarding their safe sexual behaviors). These factors can directly predict individual sexual behavior intention [20]. The TPB is widely used in behavior prediction and plays a critical role in explaining people’s behaviors [21], especially in explaining sexual behavior intentions. Lo and Huang [22] investigated college students’ sexual behavioral intentions, and the results indicated that college students’ sexual attitudes, sexual self-efficacy, and behavioral norms affect their behavioral intentions. Other studies have revealed that being a medical student does not affect sex-related outcomes. Tseng et al. [23] found that an extended TPB model could successfully predict women’s intention to have safe sex and can explain how women perceive subjective norms and gain awareness of becoming mature individuals with autonomy, which is of great importance to their sexual health. Numerous studies predicting behavioral intentions have used the TPB as the main theoretical basis.

The aforementioned literature suggests that regardless of sex education or other matters, the TPB has been widely applied to explain aspects of individual behavior [23,24,25]. Therefore, we adopted the TPB as the basic theory to explore the influence of biological sex and sexual experience on the safe sexual behavior intentions of college students.

## 3. Research Model and Hypotheses

### 3.1. Research Model

**Figure 1:**
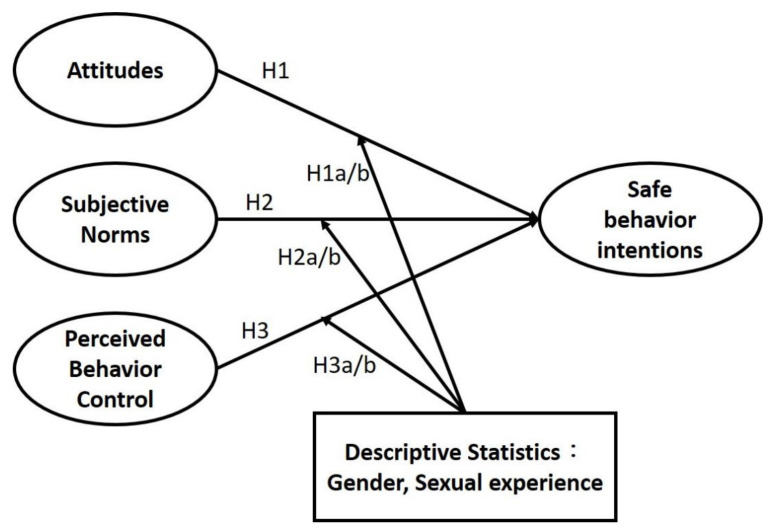
Research Model.

This study employed Ajzen’s TPB [19,21] as the theoretical basis. Because the literature has verified the effects of sexual experience and gender on sexual behavior intentions [23], this study hoped to, by extending the TPB model, explain the effects of safe sexual behaviors and more accurately predict college students’ safe sexual behavior intentions. The research structure is displayed in Figure 1.

### 3.2. Hypotheses

In the TPB, attitudes are an individual’s beliefs about behavior (e.g., sexual behavior). These beliefs are the individual’s positive or negative evaluations of the specific behavior, which affect the individual’s attitude toward the specific behavior. Therefore, the more positive the attitude is, the stronger the behavioral intention is. Studies have indicated that sexual attitudes affect students’ sexual intentions [22,26,27,28]. Therefore, we proposed H1 as follows:

H1: The sexual attitude of college students significantly influences their sexual intentions.

According to the TPB, subjective norms are directly or indirectly influenced by other individuals (e.g., family, friends, and colleagues). For example, sex education from family members and the transfer of sexual knowledge among friends or colleagues can change college students’ views on certain behaviors (e.g., sexual behavior). Research has revealed that behavioral intentions can be influenced by both subjective factors and the environment [29,30,31,32]. Therefore, we proposed H2 as follows:

H2: The subjective norms of college students significantly influence their sexual intentions.

In the TPB model, Ajzen [33] suggested that perceived behavioral control can affect behavioral intentions and directly affect actual behavior, because even if a person wants to do something, doing so requires actual behavioral control. Studies have reported that behavioral control affects students’ behavioral intentions [20,25,29]. Therefore, we proposed H3 as follows:

H3: The behavioral control of college students significantly influences their sexual intentions.

Related studies have indicated that differences in both sexual experience and biological sex influence students’ perceptions of sexual behavior [23,34]. This study posited that the behavioral intentions of college students may differ with sexual experience and biological sex. In the TPB model, biological sex and sexual experience also affect sexual intentions. Therefore, the following hypotheses were proposed:

H1 a: The sexual experience of college students affects their sexual attitude and sexual behavior intentions.

H1 b: The biological sex of college students affects their sexual attitude and sexual behavior intentions.

H2 a: The sexual experience of college students affects their subjective norms and sexual intentions.

H2 b: The biological sex of college students affects their subjective norms and sexual behavior intentions.

H3 a: The sexual experience of college students affects their behavioral control and sexual behavior intentions.

H3 b: The biological sex of college students affects their behavioral control and sexual behavior intentions.

### 3.3. Construct Operationalization

The study’s questionnaire design was adapted from the questionnaire proposed by Turchik and Gidycz [20], which investigated safe sexual behavior among US college students. The questionnaire items were contextualized to match the current situation in China. The 32 items were used to examine four main constructs. Seven, six, nine, and nine of the items on the questionnaire investigated perceived behavioral control, subjective norms, safe behavior intentions, and attitudes, respectively. The questionnaire was closely based on the scale of Turchik and Gidycz [20]. The items were measured on a 7-point Likert scale. The items were first translated from the original English into Chinese and then back into English by a professionally trained Chinese–English translator. Because the questionnaire was meant for distribution in China, translating it into Chinese aided the respondents’ understanding of the questionnaire items. The accuracy of the original translation was maximized by employing back-translation. Second, when the first draft of the questionnaire was completed, five professors with psychology- and education-related specialties were invited to revise the draft to ensure the validity and usefulness of the study. Before the questionnaire copies were distributed on the full scale, this study conducted a pretest on the questionnaire to eliminate vague or inappropriate phrasing of the questionnaire items, enhancing the questionnaire’s content validity. Given the infeasibility of conducting an in-person questionnaire survey on the street amid the COVID-19 pandemic, which was happening when this study was conducted, the authors administered the questionnaire survey to their classmates, totaling 32 participants. A reliability analysis was conducted on indicators in the questionnaire by using internal consistency, Cronbach’s α, and revealed that all constructs had a Cronbach’s α greater than the standard 0.7 [35], ranging between 0.882 and 0.923. Proven reliable, the questionnaire was then used for the full-scale survey of college students.

### 3.4. Data Collection

This study focused on the safe sexual behavior intentions of Chinese college students. Because of COVID-19 and related data collection restrictions, we employed an online questionnaire to collect data. We selected this online questionnaire approach because it enables convenient and low-cost data collection, rapid responses, and access to a wide range of users [35,36]. Because of the particularity of the topic and to ensure a high response rate, we used convenience sampling.

The reason why convenience sampling was used was that the authors are higher education teachers and the participants were higher education students. Thus, convenience sampling could more effectively select appropriate participants for the survey. To ensure the quality of the questionnaire survey, the authors distributed an online questionnaire on WeChat by first sending them to other higher education teachers or the authors’ colleagues who also worked at higher education institutions. Said colleagues then shared the online survey with teachers in other higher education institutions. Moreover, other teachers were asked to distribute the questionnaire to students they used to teach. Completion of the questionnaire was voluntary. The questionnaire was presented on WJX (https://www.wjx.cn/, accessed on 5 February 2021), a professional online questionnaire platform in China. WJX was selected because it requires participants to verify their identity through their WeChat accounts before starting the survey; this can prevent duplicate responses—namely, more than one response from one participant. Responses were collected from January to February 2021. To improve the questionnaire recovery rate, we also provided the respondents who completed the questionnaire in full with a financial incentive (RMB2–5).

The survey data were collected through WJX, and the survey page received 836 visits in total. Finally, a total of 507 questionnaires were collected through the online questionnaire system, and students from 15 universities responded. We excluded responses if any of the following were deemed true [37,38,39]: The answers for a single construct were the same (e.g., a score of 1 or 7 for all items) or the answers had extreme values; (2) the questionnaire was connected to an Internet protocol address associated with a previous response (duplicates are excluded); (3) or the response time was too short (common response times were 5–8 min; questionnaires completed in less than 3 min were regarded as invalid). We excluded 47 invalid questionnaires and used 460 valid questionnaires for formal data analysis.

In total, 278 and 182 male and female students, respectively, provided valid responses. Among the respondents, 82 were aged 18 years, 96 were aged 19 years, 120 were aged 20 years, 70 were aged 21 years, 66 were aged 22 years, and 28 were aged 23 years or above. In total 171 freshmen, 108 sophomores, 135 juniors, and 50 seniors participated. Among them, 189 had never been in love, 148 had experienced being in love, and 127 were in love during the investigation. Finally, 81 of the students had sexual experience, and 379 students had no sexual experience.

## 4. Results

The collected data were analyzed using SPSS (IBM SPSS Statistics for Windows, Version 22.0. Armonk, NY: IBM Corp.) [40] and partial least squares structural equation modeling (PLS-SEM) [40]. SPSS was used to analyze demographic data, and PLS-SEM was employed for the measurement and structural model analysis, mainly because this approach, compared with covariance-based SEM, involves analyzing the complex relationships between observed and latent variables. PLS-SEM has been widely adopted for analysis in research on marketing management, information management, organizational management, human resources management, and tourism management [41]. Finally, to mitigate common method variance, the questionnaire was deliberately paginated during data collection. This provided the respondents with sufficient rest time between pages, and the time intervals reduced the common method variance caused by the continuous use of the same rating scale [42].

### 4.1. Measurement Model

Henseler et al. [43] introduce the Standardized Root Mean Squared Residual (SRMR) as the square root of the sum of the squared differences between the model-implied and the empirical correlation matrix. A value less than 0.10 is considered a good fit [43]. In this study, a goodness of fit measure for an SRMR composite factor model was used, because our model included one formative construct. The SRMR was 0.061 for this research; these numbers indicated an acceptable model, in addition to the factor loading reliability, convergent validity, and discriminant validity of reflective indicators [44]. Based on the results of collinearity, Hair et al. [45] suggested that a variance inflation factor (VIF) below 5 indicates no collinearity problem. The results of all the constructs in this study range from 1.063 to 1.327, which shows that this study is in line with the suggested indicators in the literature. As for reliability, mainly in terms of the relationship between the reliability of the questionnaire and the accuracy of the measurement, the factor loadings have been suggested to be higher than 0.7 [45]. Based on the results of this study, the results of all factor loadings of reflective indicators are in accordance with past recommendations [46]. The reliability of internal consistency is usually assessed on the basis of two indicators: that is, the composite reliability and the Cronbach α [45]. Table 1 shows that all composite reliability values are greater than 0.70, indicating good internal consistency. The Average Variance Extracted (AVE) is an indicator of the dispersion between the statistical sampling values and the expected values, and it is recommended in the literature that the AVE should be greater than 0.5. Table 1 shows that the AVE results are greater than 0.5, which indicates a good convergent validity [46].

Discriminant validity was employed to test the ability of measurement variables to discriminate between different constructs. The square root of the average variance extracted between constructs must be greater than the correlation coefficient between constructs. Table 2 illustrates the correlation coefficient matrix between constructs, and the diagonal elements are the square roots of the average variance extracted [46]. According to Table 2, all square roots of the average variance extracted are greater than the correlation coefficients between constructs, showing that all constructs had satisfactory discriminant validity.

Henseler et al. [43] introduced the heterotrait–monotrait (HTMT) ratio of correlations, an alternative method for evaluating discriminant validity. The HTMT ratio is the value generated by comparing the average correlations between indicators across various constructs and within each construct (based on consistent loadings) [43]. If the HTMT ratio is less than 0.90, the two reflective constructs have discriminant validity. The highest HTMT value obtained in this study was 0.883. The HTMT results indicated that the model had excellent reliability and validity (Table 3).

### 4.2. Structural Model

**Figure 2:**
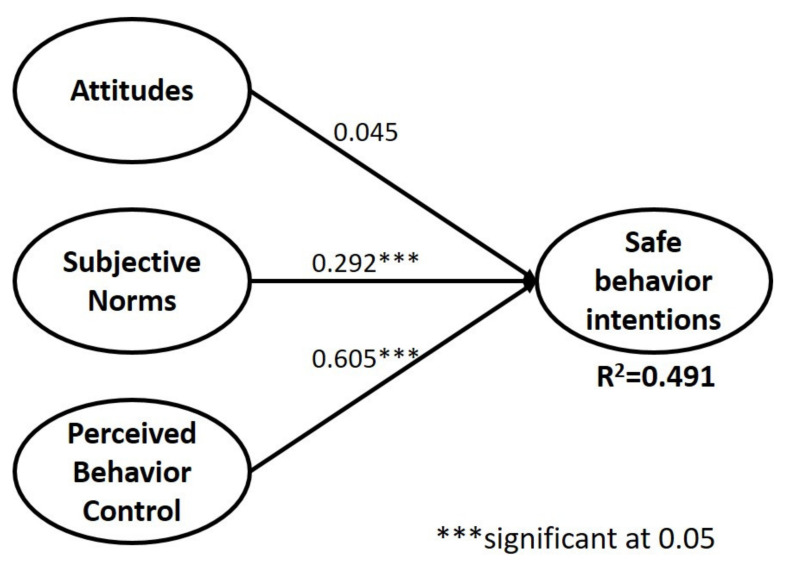
PLS results of the research model.

According to the PLS analysis results, this research used bootstrapping as the resampling method, with 5000 resamples used to assess the PLS results (Figure 2) [45]. According to the analysis results, the overall research model explained 49.1% of the variance in safe behavior intentions; thus, the current model has favorable explanatory power. For the empirical results testing H1, H2, and H3, these supported only H2 and H3. For the hypotheses related to the TPB, both subjective norms (H2) and perceived behavioral control (H3) significantly affected safe behavior intentions. Moreover, the path coefficient of perceived behavioral control reached 0.605. However, attitude (H1, β = 0.045) did not have a significant influence on safe behavior intentions.

PLS-SEM multiple-group analysis (PLS-MGA) was used to compare the two groups of samples in terms of their path coefficients in the model. The significance of the path coefficient difference was assessed according to the steps adopted by Hair et al. [45,46]. The path coefficients were compared and verified one by one. The significance of the differences in the path coefficients was based on *p* values. We compared the path coefficients between groups separated by biological sex and sexual experience. Existing studies (e.g., Chew et al., 2020; Morales et al., 2018; Potard et al., 2017) have discovered that gender and sexual experience are two factors influencing behavior. Therefore, this study used gender and sexual experience as the main classification criteria. In terms of biological sex (Table 4), only the path coefficient of behavioral control (H3 a) to behavioral intention was significantly different between the two groups (0.284**), with the coefficient being higher in men (0.626***) than in women (0.342*). The results also revealed that men had higher self-rated levels of self-control in sexual behavior than women did.

The results for sexual experience (Table 5) revealed that sexual experience had no significant effect on the results. However, when only comparing the significance of the path coefficients between the two models, we noted that the path coefficient for the subjective norms of those without sexual experience remained significant and thus merits further discussion.

## 5. Discussion

The questionnaire results were collected from 460 college students to analyze the influence of their sexual attitudes and behaviors. The results showed that there were significant differences in sexual behavior among all background variables. The proportion of male students having sexual life is higher than that of female students, which is consistent with the findings of studies in other regions of China [47,48,49,50]. According to the statistical results, 18% of the Chinese college students had engaged in sexual behavior. After further analysis and research, students aged 18–20 years had a more active sex life compared with students in other age groups. Accordingly, society has gradually become more open to the idea of sex. Schools should pay more attention to the follow-up and guiding mechanisms of sex education for college students. Currently, the education content mainly includes sex physiology education, sex psychology education, and sex ethics education. From the perspective of sexual knowledge, the fluctuation and regulation of psychological emotions in the process of sexual physiological growth, as well as the treatment of problems between men and women and the establishment of a noble personality should be considered [51]. Formally, we should establish an education system in which schools, society, and families cooperate with each other. After all, China has a traditional feudal culture and moral constraints, so the proportion of women who have sex is low, which can be speculated to be a mechanism of self-protection. In previous studies, male college students were also very concerned about whether their girlfriend was a virgin, and both sexes were very concerned about the concept of “sexual submission”. In other words, once they have sex with others, they will have a high degree of dependence on and obedience to them, which could even affect their physical and mental health. This concept has a particularly obvious impact on women [52]. Therefore, changes in sexual concepts are not influenced by just one environmental factor, but by three environmental factors.

The results indicated that the extended TPB is suitable for predicting the safe sexual behavior intentions of college students. Perceived behavioral control (H3) had the greatest influence on college students’ intention to engage in safe sexual behavior, followed by subjective norms (H2), whereas attitude (H1) had no influence. This result is consistent with the conclusions of related literature [23]. According to the aforementioned findings, to enhance the sexual health knowledge of college students, strengthening not only the concepts of subjective norms and behavioral control but also the knowledge transmission of sex education is imperative. Because subjective norms affect college students’ intentions to have safer sex, this study suggests that universities should offer relevant sex education courses to cultivate the concept of safe sex in college students. Due to the various channels through which sex-related information is transmitted, to protect college students from incorrect information, university teaching staff should guide students toward appropriate sources of sexual knowledge and help them to acquire the correct mentality regarding sexual behavior in a relationship. Moreover, Guo et al. [53] conceded that sex education courses in colleges and universities are a major challenge. This difficulty is especially apparent in the comprehensive implementation of sex education, which involves multidisciplinary knowledge and therefore requires that teachers have certain abilities. Most colleges and universities appear to have realized the importance of sex education courses, but such courses tend to be offered as elective courses. Therefore, much work is still required to popularize sex education.

In terms of biological sex, significant differences were noted in the influence of behavioral control on behavioral intentions between groups, with the effect being stronger in men than in women. However, no difference was noted between men and women in terms of subjective norms or attitudes. This contradicts the results of related studies [24,34]. Further analysis suggests that our results are attributable to male Chinese college students being more confident in their ability to be responsible for safe sex than their female counterparts. Men may be more curious to learn about sexual behavior and thus acquire more relevant knowledge and develop a stronger understanding than women. Nevertheless, the essence of safe sex is that both men and women should have adequate sexual health knowledge. Therefore, we argue that universities should place greater emphasis on courses related to sexual health education, courses related to the prevention and treatment of sexually transmitted diseases, and the awareness of safe sex; they should also continue to reinforce gender equality education and teach students to learn to protect themselves and their partners.

In discussing the TPB model, Ajzen [33] argued that perceived behavioral control can affect behavioral intentions and directly affect actual behavior. Even if individuals wish to do something, they cannot execute it without actual behavioral control. Studies have revealed that behavioral control affects students’ behavioral intentions, and the current conclusions are consistent with those of previous studies [20,27,32]. Therefore, we propose the following suggestions to enhance behavioral control: (1) promoting college students’ awareness of condoms and other contraceptive methods; (2) providing training in practical skills, such as correct condom use, tactful refusal of undesired sex, and persuasion of partners to use protection; (3) improving access to resources for female students, for example, by increasing the number of condom vending machines and enhancing privacy protection on campuses and in the community.

Subjective norms also have a positive effect on safe sexual behavior intentions. The social norms of students’ health-risk behaviors are influenced by family, peers, and the school environment [54]. Parents’ sharing of sexual health knowledge with their children is a protective factor for teenagers’ sexual and reproductive health [55]; thus, improving parents’ sexual knowledge and sex education ability merits greater attention. However, sex is a topic that is typically avoided in parent–child communication, especially in Asian countries such as China. Hyde et al. [40] highlighted that most Irish parents claim to be open about their children’s sexual behavior, but only a few communicate directly with their children regarding how to use contraception such as condoms. Parents are more likely to convey this information indirectly through innuendo and hints. The government and schools should increase the public’s awareness of youth sex education websites through various channels. Sexual health professionals should establish workshops, forums, and science-based programs for parents to help improve their ability to talk to their children about sexual behavior.

## 6. Conclusions

### 6.1. Practical Implications

According to the research results, we can understand the current state of safe sexual behavior in college students. We hope to provide suggestions for society, colleges, and college students to improve their sexual health knowledge. First, we advise society. We found that college students can obtain sex knowledge from various channels in social life, and also come into contact with a lot of incorrect information about sexual behavior. This result is similar to the results of previous studies [10]. Therefore, this study suggests the promotion of various types of safe sex knowledge through school policies. For example, schools may establish a section on their website for safe sex and sex knowledge to promote students’ understanding of and timing for using safe sex products. Such a gesture can enhance the dissemination of sex knowledge among college students and inform them of the importance of safe sex. Society should strengthen the dissemination of information regarding correct and healthy sexual concepts to provide more support for the comprehensive and healthy growth of college students. Secondly, we advise colleges and universities. School education is the main channel of sex education. Colleges and universities should pay attention to the teaching of sexual knowledge and ethics, and strengthen the normative teaching of sexual behavior. This conclusion is similar to many domestic and foreign studies [56,57]. Therefore, we suggest that colleges and universities should improve the internal sex education systems on campus, set up independent sex education courses, and increase systematic sex education knowledge. Finally, we offer suggestions to college students. Research shows that many college students are often affected by many aspects of sexual behavior, and the most important problem is that their sexual values are not correct. This result is similar to that of previous studies [58,59,60,61,62]. College students should first establish correct and healthy sexual values, know how to respect others, and understand sexual knowledge and accept sex education through formal and legal ways. The core of college sexual behavior is to follow the principles of “love”, “voluntariness” and “not harming others”.

### 6.2. Limitations and Future Research

This study adopted a questionnaire survey and convenience sampling to examine the level of sexual knowledge of contemporary Chinese college students. Therefore, our sample’s scope is limited, and it does not cover universities in all provinces of China. This limitation can be addressed in follow-up research. For example, future research may explore whether the sexual knowledge of college students in different regions differs because of disparities in the educational environment. Second, this study obtained questionnaire responses from Chinese college students, and the analysis indicated that 82.11% of these students had no sexual experience. In the age of the Internet and the related information explosion, contemporary college students should have a clear understanding of topics such as the appropriate use of and attitudes regarding condom use, as well as relevant AIDS prevention approaches. Therefore, future researchers should examine this topic from different perspectives: for example, by focusing on college students who have sexual experience in romantic relationships. Third, the need for universal sex education is not limited to college students. However, because of the high prevalence of marriage, romantic relationships, and sexual behavior among college students, this study focused on Chinese college students. Through literature research, questionnaire data observation and analysis could be based on more variables of the research subject such as age, place of domicile, marriage situation, and the sexual attitudes and sexual behavior of college students, which could be added for deeper understanding of the influences brought by these variables. Moreover, future research can explore other core factors. For example, variables such as the educational level or occupation of the guardians of the subjects can be used to effectively explain any deficiencies in the current quantitative research.

## Figures and Tables

**Table 1:** Factor Loading, Cronbach alpha, Composite Reliability & AVE.

Constructs	Items	Factor Loading	Cronbach’s Alpha	Composite Reliability	AVE
**Subjective norm**	SN1 SN2 SN3 SN4 SN5 SN6	0.857 0.889 0.915 0.914 0.872 0.883	0.947	0.957	0.79
**Safe Behavior intention**	ITU1 ITU2 ITU3 ITU4 ITU5 ITU6 ITU7 ITU8 ITU9	0.862 0.787 0.868 0.905 0.896 0.928 0.923 0.798 0.789	0.984	0.985	0.781
**Attitude**	AT1 AT2 AT3 AT4 AT5 AT6 AT7 AT8 AT9 AT10	0.838 0.922 0.906 0.894 0.89 0.894 0.861 0.901 0.891 0.879	0.957	0.963	0.746
**Perceived behavior control**	PBC1 PBC2 PBC3 PBC4 PBC5 PBC6 PBC7	0.817 0.93 0.926 0.955 0.962 0.881 0.866	0.963	0.97	0.822

Notes: Perceived behavior control (PBC), Attitude (AT), Safe behavior intention (ITU), Subjective norm (SN).

**Table 2:** Analysis of discriminant validity (Fornell–Larcker criterion).

	Subjective Norm	Attitude	Safe Behavior Intention	Perceived Behavior Control
Subjective norm	0.889			
Attitude	0.101	0.884		
Safe Behavior intention	0.526	0.198	0.863	
Perceived behavior control	0.455	0.244	0.649	0.907

**Table 3:** Analysis of discriminant validity (Heterotrait-Monotrait Ratio).

	Subjective Norm	Attitude	Safe Behavior Intention	Perceived Behavior Control
Subjective norm				
Attitude	0.1			
Safe Behavior intention	0.543	0.206		
Perceived behavior control	0.474	0.246	0.668	

**Table 4:** Result of PLS-MGA (Gender).

Hypotheses	Relationship	Path Coefficients of Boy	Path Coefficients of Girl	Path Coefficients -Diff	*p*-Value
H1/1 a	Attitude	0.041	0.078	−0.039	0.639
H2/2 a	Subjective norm	0.212 ***	0.353 ***	−0.14	0.201
H3/3 a	Perceived behavior control	0.626 ***	0.342 ***	0.284	0.033 **

Notes: ** *p* < 0.05; *** *p* < 0.01.

**Table 5:** Result of PLS-MGA (sex experience).

Hypotheses	Relationship	Path Coefficients of Sex Experience	Path Coefficients of No Sex Experience	Path Coefficients -Diff	*p*-Value
H1/1 a	Attitude	0.044	0.039	0.005	0.891
H2/2 a	Subjective norm	0.285	0.292 ***	−0.007	0.985
H3/3 a	Perceived behavior control	0.447 ***	0.533 ***	−0.089	0.624

Notes: *** *p* < 0.01.

## Data Availability

Not applicable.
